# Genomic insights into the genetic structure and population history of Mongolians in Liaoning Province

**DOI:** 10.3389/fgene.2022.947758

**Published:** 2022-10-12

**Authors:** Xuwei Hou, Xianpeng Zhang, Xin Li, Ting Huang, Wenhui Li, Hailong Zhang, He Huang, Youfeng Wen

**Affiliations:** Institute of Biological Anthropology, Jinzhou Medical University, Jinzhou, China

**Keywords:** Mongolian, genetic structure, population admixture, Liaoning Province in China, genome-wide data

## Abstract

The Mongolian population exceeds six million and is the largest population among the Mongolic speakers in China. However, the genetic structure and admixture history of the Mongolians are still unclear due to the limited number of samples and lower coverage of single-nucleotide polymorphism (SNP). In this study, we genotyped genome-wide data of over 700,000 SNPs in 38 Mongolian individuals from Fuxin in Liaoning Province to explore the genetic structure and population history based on typical and advanced population genetic analysis methods [principal component analysis (PCA), admixture, F_ST_, *f*_*3*_-statistics, *f*_*4*_-statistics, *qp*Adm/*qp*Wave, *qp*Graph, ALDER, and TreeMix]. We found that Fuxin Mongolians had a close genetic relationship with Han people, northern Mongolians, other Mongolic speakers, and Tungusic speakers in East Asia. Also, we found that Neolithic millet farmers in the Yellow River Basin and West Liao River Basin and Neolithic hunter–gatherers in the Mongolian Plateau and Amur River Basin were the dominant ancestral sources, and there were additional gene flows related to Eurasian Steppe pastoralists and Neolithic Iranian farmers in the gene pool of Fuxin Mongolians. These results shed light on dynamic demographic history, complex population admixture, and multiple sources of genetic diversity in Fuxin Mongolians.

## Introduction

Northeast Asia is a vast geographical region encompassing the Mongolian Plateau (MP), Yellow River Basin (YRB), West Liao River Basin (WLRB), Amur River Basin (ARB), Russian Far East, Korean Peninsula, and Japanese Islands. Recent studies indicated that frequent and complex population migration, exchange, and admixture events had happened in this region. The West Liao River Basin is considered the cradle of the Transeurasian language family. The “Transeurasian hypothesis” is supported by evidence from linguistics, archaeology, and genetics. It indicates that Japonic, Koreanic, Tungusic, Mongolic, and Turkic languages are split from a proto-Transeurasian language, and the diffusion of Transeurasian language is related to the expansion of early millet farmers in the West Liao River Basin (Robbeets et al., 2021), but Wang et al. (2021a) hold opposite views: they did not find the West Liao River farmer-related ancestry in ancient populations in the Mongolian Plateau and Amur River Basin. The Yellow River Basin is considered the origin of the Sino-Tibetan language from different perspectives such as archaeology, genetics, and linguistics. It supports the “northern-origin hypothesis,” and the diffusion of the Sino-Tibetan language also is considered to conform “farming–language dispersal hypothesis” that the Neolithic YRB millet farmers who are related to Yangshao and/or Majiayao cultures may be the ancestors of Sino-Tibetan language speakers (Sagart et al., 2019; Zhang et al., 2019; Wang et al., 2021a). Paleogenomic research studies showed that there is up to 14,000-year genetic continuity from ancient ARB population to modern Tungusic people, and modern Tungusic people show genetic homogeneity with each other (He et al., 2021; Mao et al., 2021; Wang et al., 2021a), but there is an exception in Tungusic speakers that Manchu people exhibit significant genetic similarity with northern Han people (Zhang et al., 2021b). The Eurasian Steppe zone is the largest steppe zone in the world that stretches from Eastern Europe through Mongolia to Northeast China, Bronze-Age Yamnaya Steppe pastoralists expanded eastward through the Eurasian Steppe, and then Afanasievo, Andronovo, and Sintashta cultures, which are related to the Yamnaya culture, established, respectively (Haak et al., 2015; de Barros Damgaard et al., 2018; Ning et al., 2019). Steppe pastoralists have a genetic influence on East Asian populations, but their genetic contributions are limited (Ning et al., 2019; Wang et al., 2021a; Yang et al., 2021). The influence of steppe pastoralists on the genetic formation of MP populations was discontinuous (Wang et al., 2021a), but the steppe pastoralist-related ancestry has persisted in Northwest China since the Early Bronze Age (∼3000 BCE) (Ning et al., 2019; Wang et al., 2021c; Zhang et al., 2021a), and the steppe pastoralist-related ancestry also exists in the genetic makeup of modern populations in South Siberia and Western and Northern China (Feng et al., 2017; He et al., 2021; Ma et al., 2021). In general, previous studies have found dynamic demographic history and multiple sources of genetic diversity in Northeast Asia, but the genetic structure and affinity of modern populations in Northeast Asia remain unclear due to a limited number of samples.

**FIGURE 1:**
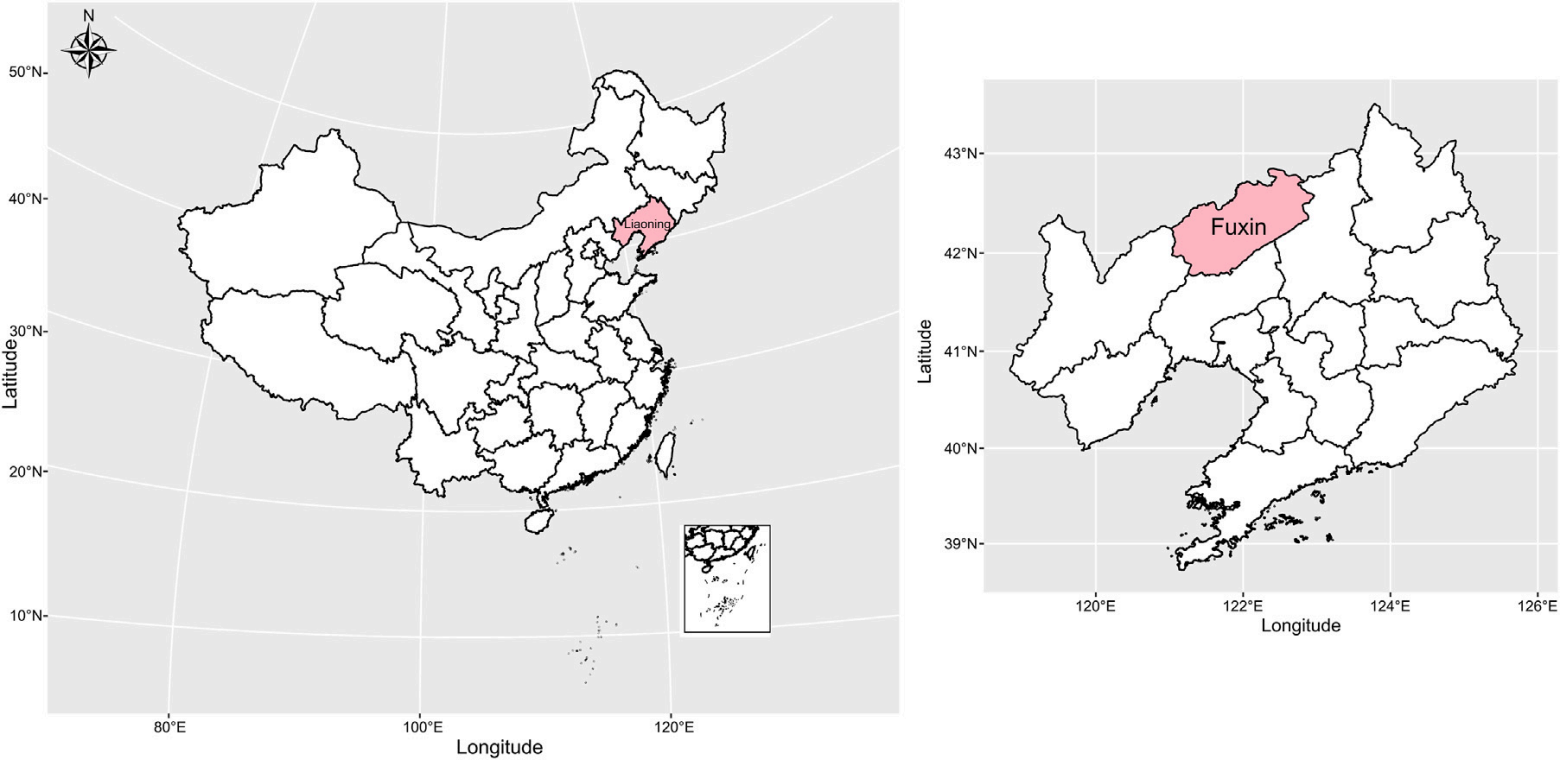
Geographical positions of the sampling population in this study.

Mongolians play an indispensable role in the formation of culture and genetic structure in Eurasia, the Mongol Empire controlled vast territories and trade routes stretching from East Asia to Europe in the 13th century, and there are genetic imprints of Mongolians which can be found in modern Eurasian populations (Dulik et al., 2011; Bai et al., 2014; Bai et al., 2018; Jeong et al., 2020). The Mongolian population exceeds six million and is the largest population among the Mongolic speakers in China. Modern Mongolians are widely distributed in China, but they mainly live in Inner Mongolia, Liaoning, Xinjiang, and other northern provinces. Chinese Mongolians from different regions have different genetic profiles, northern Mongolians possess significant genetic contribution from ancient ARB populations, southern Mongolians possess a majority of Neolithic YRB farmer-related ancestry, and Guizhou Mongolians harbor more southern ancestry related to Tai–Kadai, Austroasiatic, and Austronesian speakers. This reveals Mongolians gradually mixed with the local indigenous people along with their migration (Chen et al., 2021; He et al., 2021). Also, there is a different admixture history in western and eastern Chinese Mongolians, the western Mongolians receive more genetic influence of western Eurasians, and the Eastern Mongolians possess more Neolithic YRB- and ARB-related ancestry (He et al., 2021; Yang et al., 2021). Ancient Mongolia is formed by multiple tribes, every ancient Mongolian tribe experience a different origin, exchange, and admixture history, and their descendants live in different regions, which may be one of the reasons why genetic differences existed in modern Mongolians. “Tumet” tribe is a larger Mongolian tribe and has a controversial origin; it has been divided into two groups (Western Tumet group and Eastern Tumet group) according to geographical distribution since the 17th century. The Western Tumet group is mainly distributed in the Inner Mongolian Autonomous Region (Hohhot and Baotou), and the Eastern Tumet group is distributed in Liaoning Province (Fuxin and Chaoyang). Previous studies based on genome-wide data have reported the genetic structure and admixture history of Baotou Mongolians who are descendants of the Western Tumet group, but the genetic structure and admixture history of Liaoning Mongolians is still ambiguous. In this study, we generated genome-wide data of over 700,000 SNPs in 38 Mongolian individuals from Fuxin in Liaoning Province (Figure 1) and combined all available modern and ancient East Asian populations to investigate 1) the genetic profile and structure of Fuxin Mongolians; 2) genetic differences of Chinese Mongolians in different regions; 3) genetic affinity between Fuxin Mongolians and Han people, Tungusic speakers, and Mongolic speakers; 4) how many ancestral sources contributed to Fuxin Mongolians; and 5) to shed light on the proportions of the genetic contribution of the millet farmers in Yellow River Basin and West Liao River Basin, Northern Asian hunter–gatherers, and Western Eurasian populations.

## Materials and methods

### Sampling and genotyping

In this study, we collected peripheral blood samples from 38 Mongolian individuals (15 male and 23 female) in Fuxin, Liaoning Province, China. The detailed sample information is listed in Supplementary Table S1. Every participant signed the written informed consent before the study begins. The geographical position of the sampling population in this study is shown in Figure 1. All participants were required to be indigenous residents whose ancestors have lived in the sampling site for at least three generations, and self-declared ethnicity and family migration history were recorded. This project was reviewed and approved by the Medical Ethics Committee of Jinzhou Medical University (JZMULL2021101), and all procedures were carried out in accordance with the recommendations of the 2,000 Helsinki Declaration (Helsinki, 2001). DNA extraction was performed by using the Genomic DNA Extraction Kit following manufacturer’s instructions, and all DNA samples were genotyped using Illumina WeGene V3 Arrays. The raw data contained 717,228 single-nucleotide polymorphisms (SNPs) and were filtered using PLINK 1.9 (Purcell et al., 2007) based on the predefined threshold (-maf: 0.01, -hwe: 0.0001, -mind: 0.01, and -geno: 0.01). Also, we applied Genome-wide Complex Trait Analysis (GCTA) (Yang et al., 2011) software to estimate the genetic relationship between newly sampled Mongolian individuals, and the individual who has relatedness up to 0.125 (third-degree relative) with other newly sampled individuals was removed to guarantee studied individuals were unrelated (Supplementary Table S2; Supplementary Figure S1). Finally, we obtained a dataset containing 36 individuals with 477,492 SNPs which was used to perform the following population genetic analysis.

### Data merging

In this study, we merged our newly genotyped 36 individuals’ data with the Affymetrix Human Origins (HO) array dataset (Patterson et al., 2012) and recently published population data from Baotou Mongolian and Bijie Mongolian (Chen et al., 2021; Yang et al., 2021) to constitute a dataset containing 52,403 SNPs that was used to perform principal component analysis (PCA), F_ST_, admixture, TreeMix, and ALDER. Also, we merged newly genotyped data with the 1240K dataset from the Reich Lab (https://reich.hms.harvard.edu/allen-ancient-dna-resource-aadr-downloadable-genotypes-present-day-and-ancient-dna-data) to obtain a dataset with a larger number of SNPs (139,592 SNPs) for performing *f*_3_-statistics, *f*_4_-statistics, *qp*Wave/*qp*Adm, and *qp*Graph.

### Principal component analysis and ADMIXTURE

In this study, PCA and admixture were used to explore the population structure in Eurasia. First, we performed the PCA based on the merged-HO dataset containing 52,403 SNPs from 1,488 individuals in 151 populations using the smartpca program in EIGENSOFT software (Patterson et al., 2006) with the following parameters: numoutlieriter: 0 and lsqproject: YES, and the visualization was carried out using R software, the background of 2-D plots was constructed based on modern populations, and all ancient populations were projected onto it. Next, we applied PLINK 1.9 (Purcell et al., 2007) with the parameters “-indep-pairwise 200 25 0.4” to remove SNPs in strong linkage disequilibrium, and then we performed admixture analysis, which is a model-based clustering analysis (Alexander et al., 2009), with a predefined number of ancestral sources (K) ranging from 2 to 20. Also, the admixture analysis was also performed based on a merged-HO dataset, which contained 2,123 individuals from 179 populations. An optimal value of ancestral sources (K) was selected using the 10-fold cross-validation (CV) errors, which are listed in Supplementary Table S3. The visualization of admixture results was carried out using AncestryPainter (Feng et al., 2018) and R package pophelper.

### Pairwise F_ST_ genetic distances

We used the smartpca program in EIGENSOFT software (Patterson et al., 2006) with parameters: fstonly: YES to calculate pairwise F_ST_ genetic distances between Fuxin Mongolian and other modern reference populations. The heatmap and phylogenetic tree were used to shed light on the relationship between Fuxin Mongolians and other populations. The heatmap was plotted using the R package pheatmap, the neighbor-joining (N-J) tree was constructed using MEGA X (Kumar et al., 2018), and the visualization was performed using the online tool iTOL (https://itol.embl.de/).

### *F*-statistics

All *f-*statistics were carried out using ADMIXTOOLS (Patterson et al., 2012). First, we applied qp3pop in ADMIXTOOLS (Patterson et al., 2012) with default parameters to perform the three-population test (*f*_3_-statistics). We calculated outgroup-*f*_*3*_ (Mongolian_Fuxin, Y; Yoruba) to measure shared genetic drifts between Fuxin Mongolians and reference populations (Y) and computed admixture-*f*_*3*_ (X, Y; Mongolian_Fuxin) to explore the potential admixture signals. Also, we then performed the four-population test (*f*_*4*_-statistics) using the qpDstat program in ADMIXTOOLS (Patterson et al., 2012) with default parameters to examine shared alleles and infer the direction of the gene flow.

### *qp*Adm/*qp*Wave and *qp*Graph

We used *qp*Wave/*qp*Adm programs in ADMIXTOOLS (Patterson et al., 2012) to determine the minimum number of ancestral sources and quantify the ancestral proportion. In this study, we used the following ten outgroups, namely, Mbuti, Malaysia_LN, Tianyuan, Papuan, Ust_Ishim, GreatAndaman, Kostenki14, Australian, Mixe, and Atayal to test two-way admixture models. Also, we used the *qp*Graph program in ADMIXTOOLS (Patterson et al., 2012) to explore the best fitting phylogenetic framework with population splits and gene flow events and reconstruct the deep population history of Fuxin Mongolians.

### TreeMix and ALDER

To further explore the relationship between Fuxin Mongolians and other reference populations, we applied TreeMix v1.13 software (Pickrell and Pritchard, 2012) to construct a rooted maximum likelihood tree with gene flow events varying from 0 to 10, and the best-fitted models were chosen based on the predefined hypothesis and residual values. The admixture time and possible ancestral sources of Fuxin Mongolians were estimated by using multiple admixture-induced linkage disequilibrium for evolutionary relationships (ALDER) (Loh et al., 2013).

### Y chromosomal and mtDNA lineages

We used an in-house script to assign the Y-chromosomal and mitochondrial haplogroups following the recommendations of the International Society of Genetic Genealogy (ISOGG; http://www.isogg.org/) and mtDNA PhyloTree17 (http://www.phylotree.org/). The haplogroup information of mtDNA and Y chromosome is listed in Supplementary Table S11.

## Results

### Population genetic structure

**FIGURE 2:**
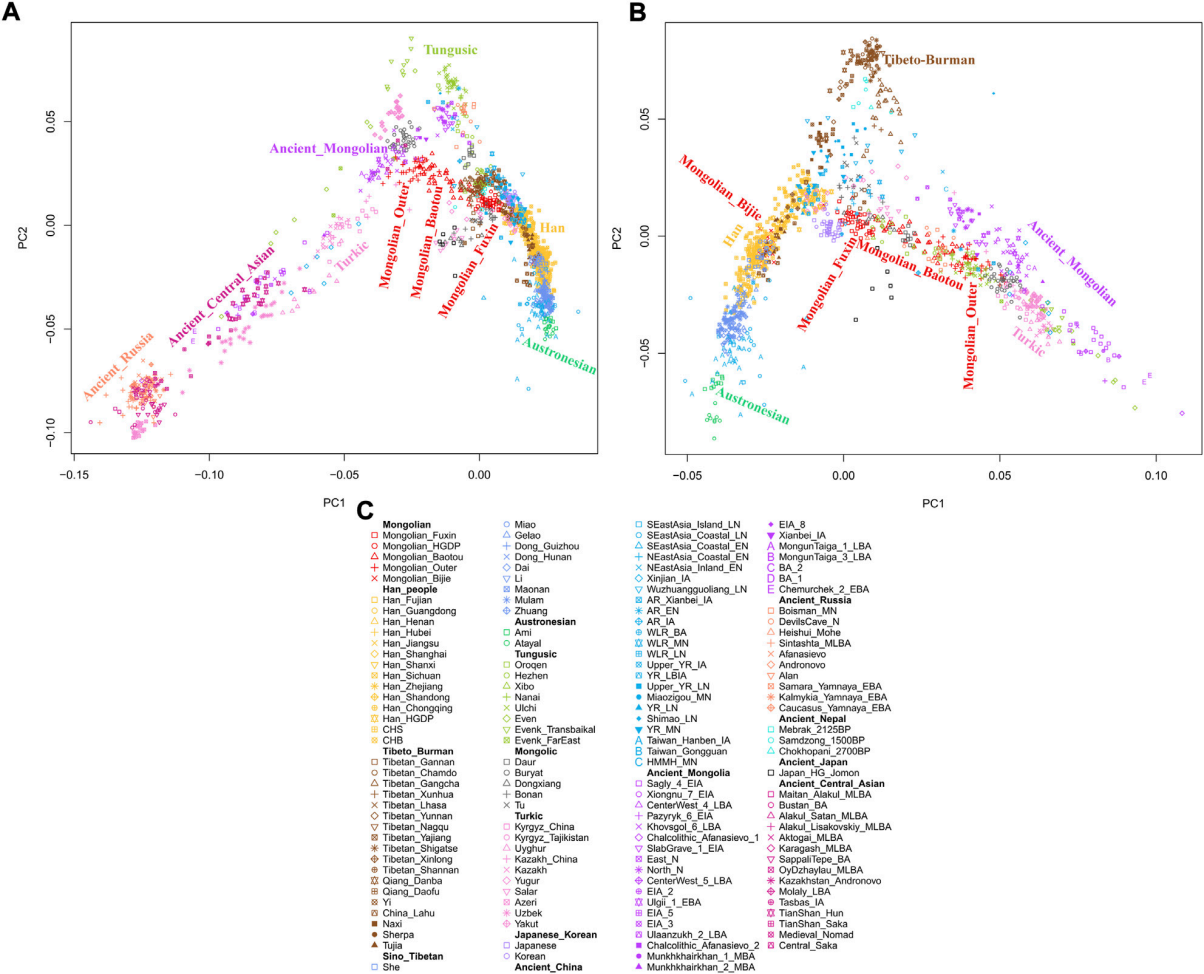
The population structure of modern and ancient Eurasian populations inferred by principal component analysis based on genome-wide data. The color of modern population was consistent with language categories, and all ancient populations were projected onto it. **(A∼B)** PCA results showed an overview population relationship between Fuxin Mongolians and other modern and ancient populations. **(C)** Population list of PCA.

**FIGURE 3:**
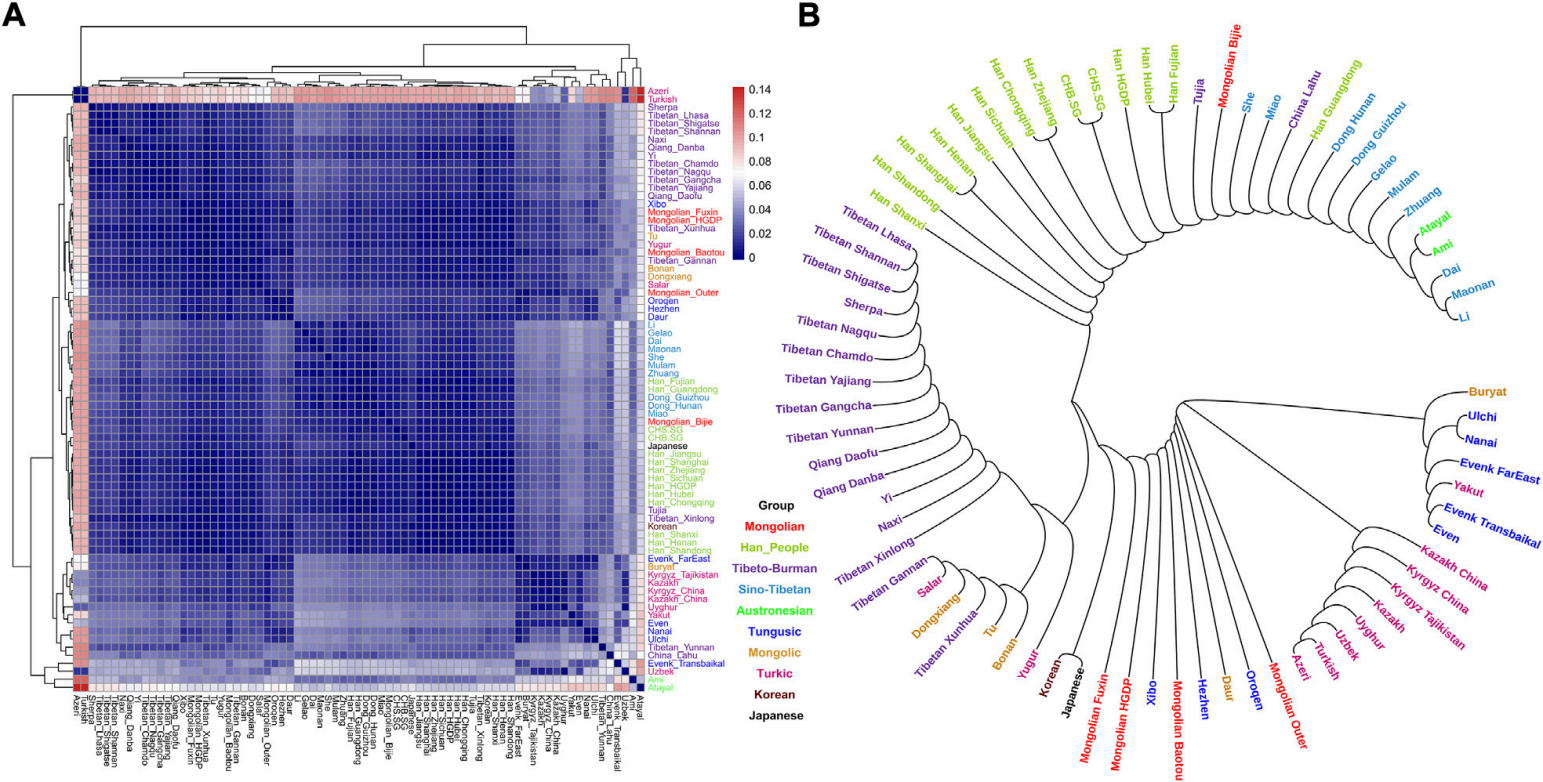
Results of pairwise FST among modern East Asian populations. Each population was marked by different colors which was corresponded to their language category. **(A)** Heatmap showed the pairwise FST genetic distance. **(B)** Neighbor-joining (NJ) phylogenetic tree showed the genetic relationship between Fuxin Mongolians and other modern East Asians.

**FIGURE 4:**
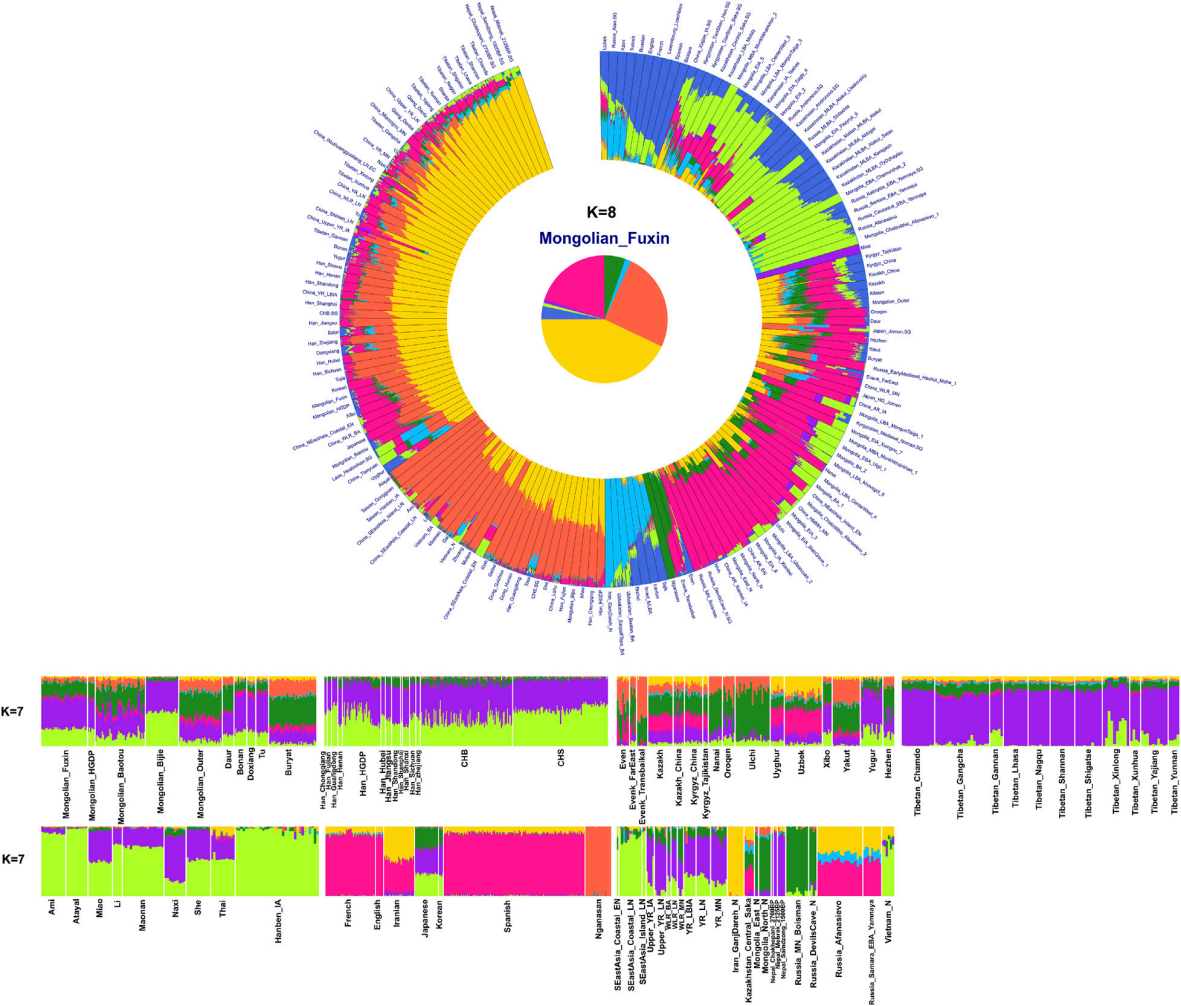
Results of model-based admixture clustering analysis showed the ancestral composition of studied Mongolians and reference populations at *K* = 7 and *K* = 8.

To explore the general patterns of genetic structure between Fuxin Mongolians and other Eurasian reference populations, we conducted PCA first. There were obvious genetic clusters such as Han cline, Mongolian cline, and Turkic speaker cline that could be observed which were consistent with their language categories and geographical distribution (Figure 2). This studied Mongolian people were surrounded by northern East Asian populations such as Han people, Tungusic speakers, Turkic speakers, Mongolic speakers, Tibeto-Burman speakers, and Japanese as well as Korean. We also found some ancient YRB and WLRB populations which were close to Fuxin Mongolians. There were obvious genetic differences between Fuxin Mongolians and Bijie Mongolians, modern Tai–Kadai, Hmong–Mien, and Austronesian speakers as well as ancient populations in Central Asia and Russia. Four present-day northern Mongolians formed a cluster, and their positions in Figure 2 were consistent with geographic distribution. Fuxin Mongolians were close to Hulunbuir Mongolians, Baotou Mongolians were located between Outer Mongolians and Mongolians from Fuxin and Hulunbuir, and Outer Mongolians were adjacent to ancient Mongolians. The same genetic structure could be observed in the results of pairwise-F_ST_ analysis, and we found people who belonged to the same language category always clustered together in East Asian populations. Fuxin Mongolians had a close genetic affinity with Hulunbuir Mongolians, and there were close genetic distances between Fuxin Mongolians and Han people (FST_Shanxi_ = 0.001 and FST _Henan_ = 0.001), Baotou Mongolian (0.002), Xibo (0.002), Tu (0.002), and Korean (0.002) (Figure 3, Supplementary Table S4). In the model-based admixture clustering analysis, we found that when *K* = 8, the CV error was the lowest (Supplementary Table S3). The results showed that there were four dominant ancestral components which were marked in yellow, orange, pink, and deep green color in the genetic makeup of Fuxin Mongolians (Figure 4). The yellow ancestry was maximized in the hunter–gatherers from Nepal, it was also enriched in millet farmers from the Yellow River Basin, and it possessed the main proportion in the genetic makeup of modern Tibetans and Han people. The orange ancestry was maximized in the Iron-Age Hanben population from Taiwan and Neolithic southern populations in Fujian, and it also commonly existed in modern Tai–Kadai, Hmong–Mien, and Austronesian speakers. The pink ancestry was maximized in hunter–gatherers from the Amur River Basin and Mongolian Plateau such as Boisman, Devilscave, Mongolia_North_N, and Mongolia_East_N, and it also possessed a dominant proportion in the genetic makeup of ancient Mongolians. In modern populations, the pink ancestry was enriched in Tungusic-speaking populations such as Ulchi and Nanai. The deep green ancestry was maximized in indigenous Nganasan in Siberia and prevalent in northern East Asian populations. In addition to the aforementioned four ancestral components, there were other three ancestral compositions in the genetic makeup of Fuxin Mongolians which were enriched in Western Eurasians, Eurasian Steppe pastoralists, and Central Asian populations, respectively. We found that the genetic profile of Fuxin Mongolians was similar to Hulunbuir Mongolians, but there were more Sino-Tibetan-related ancestry and southern ancestry and less genetic contribution of populations in the Amur River Basin, Siberia, and Mongolian Plateau, relative to Baotou Mongolians and Outer Mongolians. When *K* = 7 (Figure 4), the results were consistent with the aforementioned description, and the YRB farmers and Nepal hunter–gatherers, ARB and MP hunter–gatherers, and southern East Asian populations were the dominant ancestral contributors to Fuxin Mongolians.

### *f*_*3*_- and *f*_*4*_-statistics

**FIGURE 5:**
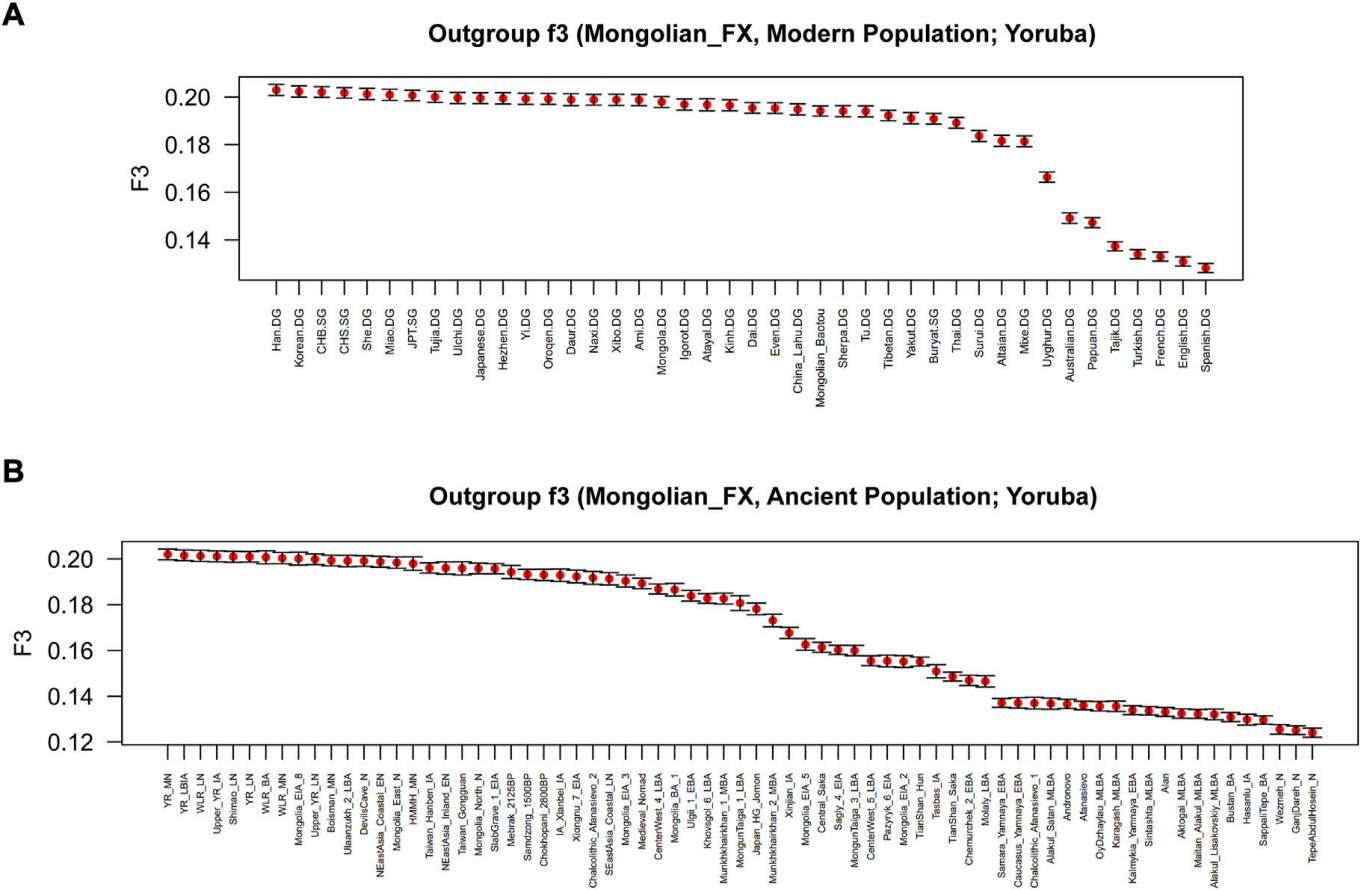
Shared genetic drift estimated *via f*_3_-statistics of the form *f*_3_ (Mongolian_Fuxin, Y; Yoruba) based on the merged 1240K dataset. **(A)** outgroup-*f*_3_ (Mongolian_Fuxin, Modern population; Yoruba). **(B)** outgroup-*f*_3_ (Mongolian_Fuxin, Ancient population; Yoruba).

**FIGURE 6:**
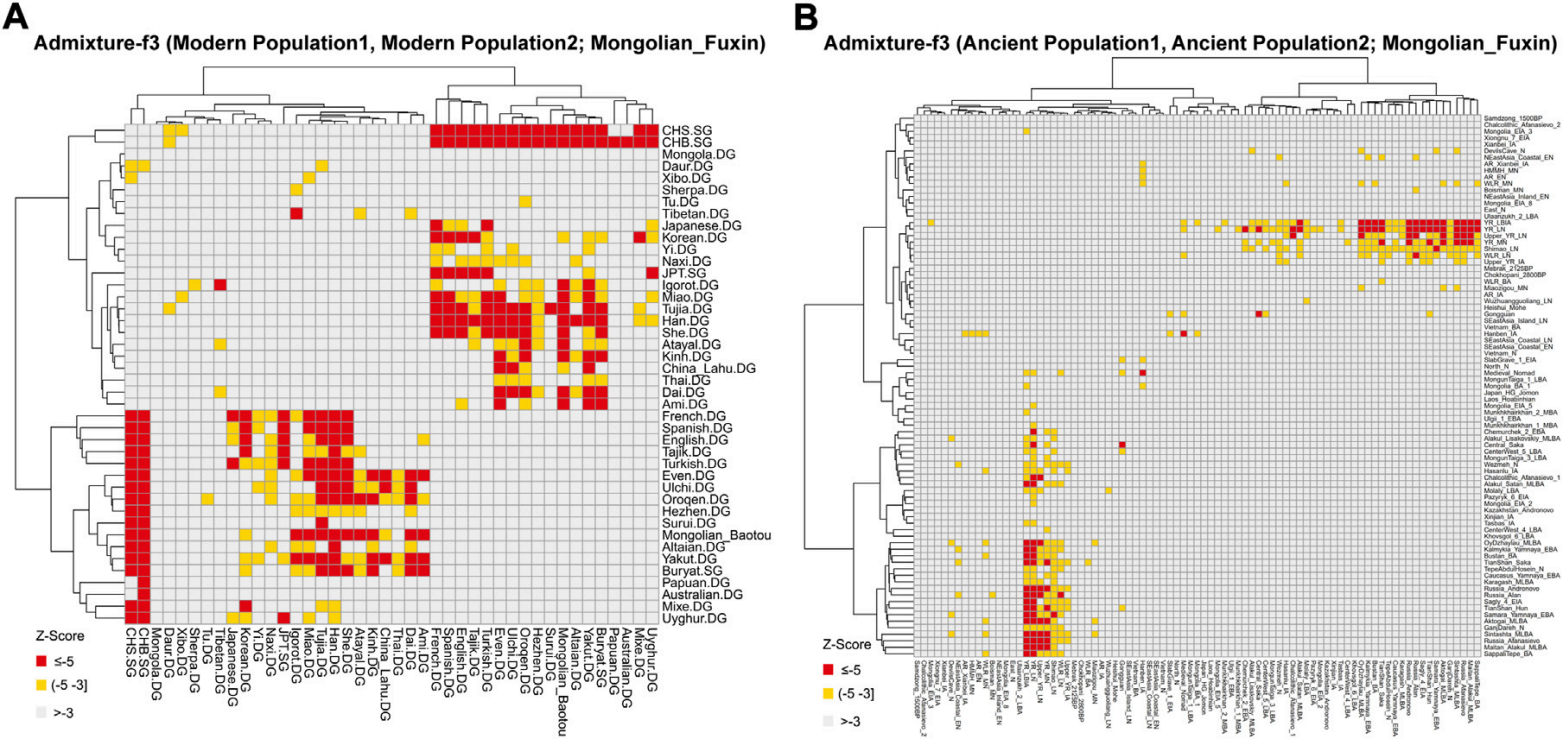
Heatmap of the admixture *f*_3_-statistics of the form *f*_3_ (X, Y; Mongolian_Fuxin). The red and yellow color indicated significant admixture signals, and X and Y were the possible ancestral contributors of Fuxin Mongolians. **(A)** Admixture-*f*_3_ (Modern population1, Modern population2; Mongolian_Fuxin). **(B)** Admixture-*f*_3_ (Ancient population1, Ancient population2; Mongolian_Fuxin).

To further test the genetic relationship observed in PCA, FST, and admixture, we performed outgroup-*f*_*3*_ and admixture-*f*_*3*_ statistics to examine shared genetic drifts and explore potential admixture signals between Fuxin Mongolians and other reference populations. In outgroup-*f*_*3*_ (Mongolian_Fuxin, Y; Yoruba) (Figure 5 and Supplementary Tables S5A,B), we found that Fuxin Mongolians showed obvious genetic similarity with Han people, and Fuxin Mongolians also shared more genetic drifts with Korean, Japanese, She, Miao, Tujia, Ulchi, Hezhen, Oroqen, and Daur. When Y represented ancient populations, we found that Fuxin Mongolians shared more genetic drifts with ancient YRB, WLRB, ARB, and MP populations. We next carried out admixture-*f*_*3*_ (X, Y; Mongolian_Fuxin) (Figure 6; Supplementary Tables S5C,D) to model possible admixture. When X and Y represented modern populations, we found significant negative Z scores (Z < −3) between Han people and Tungusic speakers (Hezhen, Oroqen, and Ulchi), Turkic speakers (Uyghur, Turkish, and Altaian), Mongolic speakers (Baotou Mongolian and Buryat), and Western Eurasian populations (Spanish, French, and English). Also, we found negative Z scores when X was ancient YRB populations from the Neolithic to Iron Age and Y represented ancient populations in Eurasian Steppe, Central Asia, and Mongolian Plateau.

**FIGURE 7:**
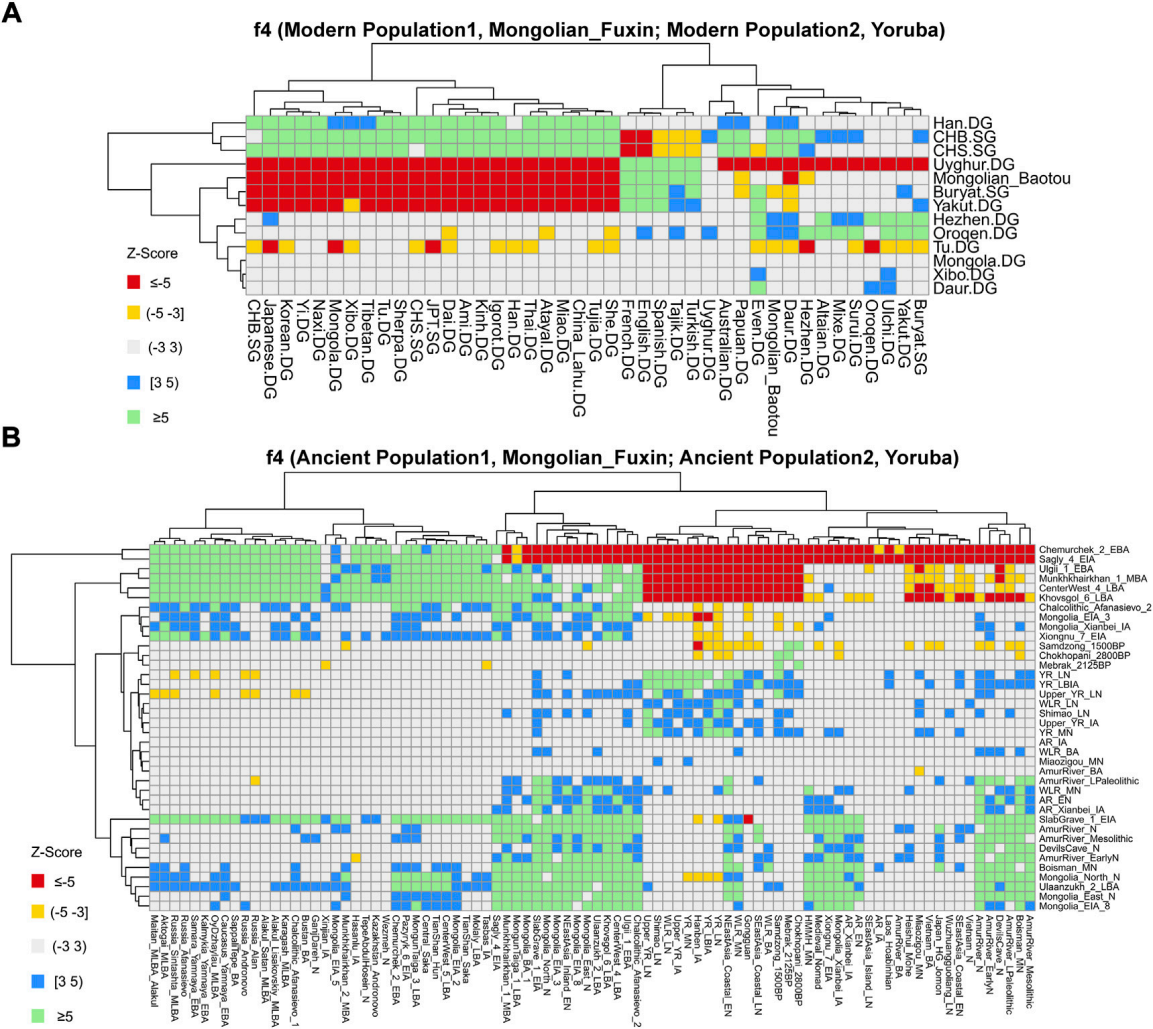
Results of *f*_*4*_-statistics performed in the form of **(A)** *f*_*4*_ (Modern Population1, Mongolian_Fuxin; Modern Population2, Yoruba); **(B)** *f*_*4*_ (Ancient Population1, Mongolian_Fuxin; Ancient Population2, Yoruba) exhibited the genetic affinity between Fuxin Mongolians and possible ancestral contributors.

We then performed *f*_*4*_-statistics to explore the asymmetric genetic relationship and gene flow direction between Fuxin Mongolians and other modern/ancient populations in the forms of *f*_*4*_ (X, Y; Mongolian_Fuxin, Yoruba) and *f*_*4*_ (X, Mongolian_Fuxin; Y, Yoruba). In the form of *f*_*4*_ (X, Y; Mongolian_Fuxin, Yoruba) (Supplementary Table S6), we observed significant negative *f*_*4*_ values when X was Han people and Y represented other modern populations, and it revealed that Fuxin Mongolians shared the most alleles with Han people relative to other modern Eurasian populations. We also found that Fuxin Mongolians shared more alleles with Hulunbuir Mongolians than Baotou Mongolians, and Daur, Hezhen, Oroqen, Ulchi, and Xibo also shared more alleles with Fuxin Mongolian than Baotou Mongolians. We next performed the *f*_*4*_-statistic in the form of *f*_*4*_ (Modern Population1, Mongolian_Fuxin; Modern Population2, Yoruba) (Figure 7; Supplementary Table S7), and the results further indicated that Han, Hulunbuir Mongolian, Xibo, Hezhen, and Daur shared more alleles with Fuxin Mongolian than other modern populations. However, the Han people exhibited a closer genetic relationship with southern East Asian populations, Xibo, Hezhen, and Daur shared more alleles with Tungusic-speaking populations relative to Fuxin Mongolians, and Hulunbuir Mongolians had the closest genetic affinity with Fuxin Mongolians. When X and Y represented ancient populations in the forms of *f*_*4*_ (X, Y; Mongolian_Fuxin, Yoruba), we found Fuxin Mongolians shared more alleles with ancient YRB, WLRB, ARB, and MP populations (Supplementary Table S6), and the results of *f*_*4*_-statistic in the form of *f*_*4*_ (Ancient Population1, Mongolian_Fuxin; Ancient Population2, Yoruba) (Figure 7; Supplementary Table S8) further revealed that there were significant genetic contributions in Fuxin Mongolians from ancient YRB populations related to the Neolithic Yangshao, Miaozigou, and Shimao culture, ancient WLRB populations related to the Hongshan, Lower Xiajiadian, and Upper Xiajiadian culture, Neolithic ARB and MP hunter–gatherers, Bronze-Age Ulaanzukh culture pastoralists (1,448–1,292 calBCE), and Iron-Age Mongolia_EIA_8 individuals (971–830 calBCE).

### *qp*Wave/*qp*Adm

**FIGURE 8:**
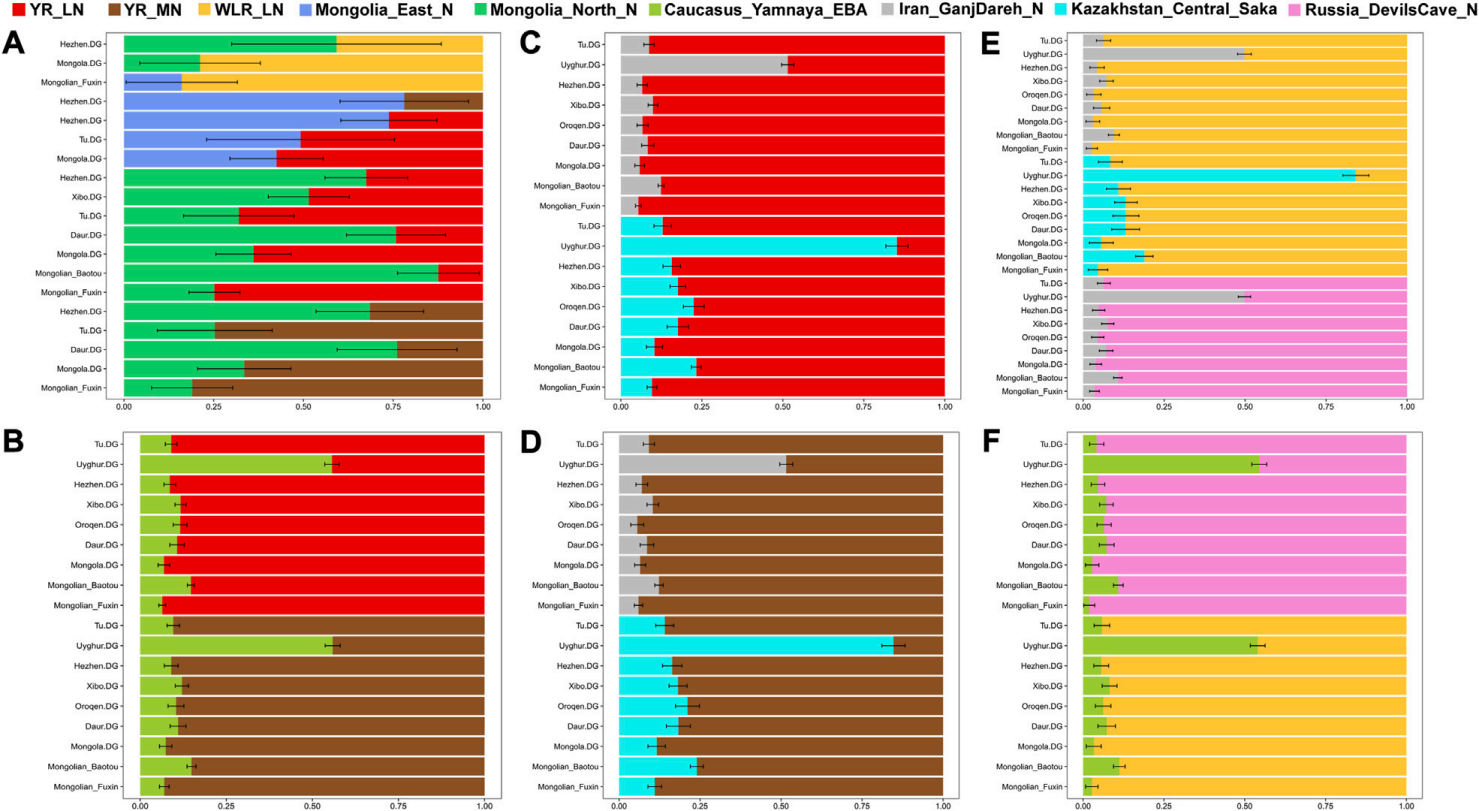
*qp*Wave/*qp*Adm results showed the detailed ancestral composition in the best‐fitted two‐way admixture model. **(A∼F)** Different models showed discrepant ancestral composition among Mongolic, Tungusic and Turkic speakers.

We applied *qp*Wave/*qp*Adm methods to explore the possible ancestral contributors and estimate their admixture proportions in Fuxin Mongolians. We used millet farmers related to Neolithic Yangshao, Longshan, and Lower Xiajiadian cultures, Neolithic hunter–gatherers in the Amur River Basin and Mongolian Plateau, Iranian Neolithic farmers, Bronze-Age Yamnaya pastoralists, and Bronze-Age Sake peoples in Central Asia as the possible ancestral sources (Figure 8; Supplementary Table S9). We found that the main genetic contributions in Fuxin Mongolians were from ancient populations in the Yellow River Basin, West Liao River Basin, Amur River Basin, and Mongolian Plateau, and there were additional gene flows related to Western Eurasian and Central Asian populations. Also, relative to other Mongolic, Tungusic, and Turkic speakers, Fuxin Mongolians had more millet farmer-related ancestry and ARB hunter–gatherers-related ancestry.

### *qp*Graph and TreeMix

**FIGURE 9:**
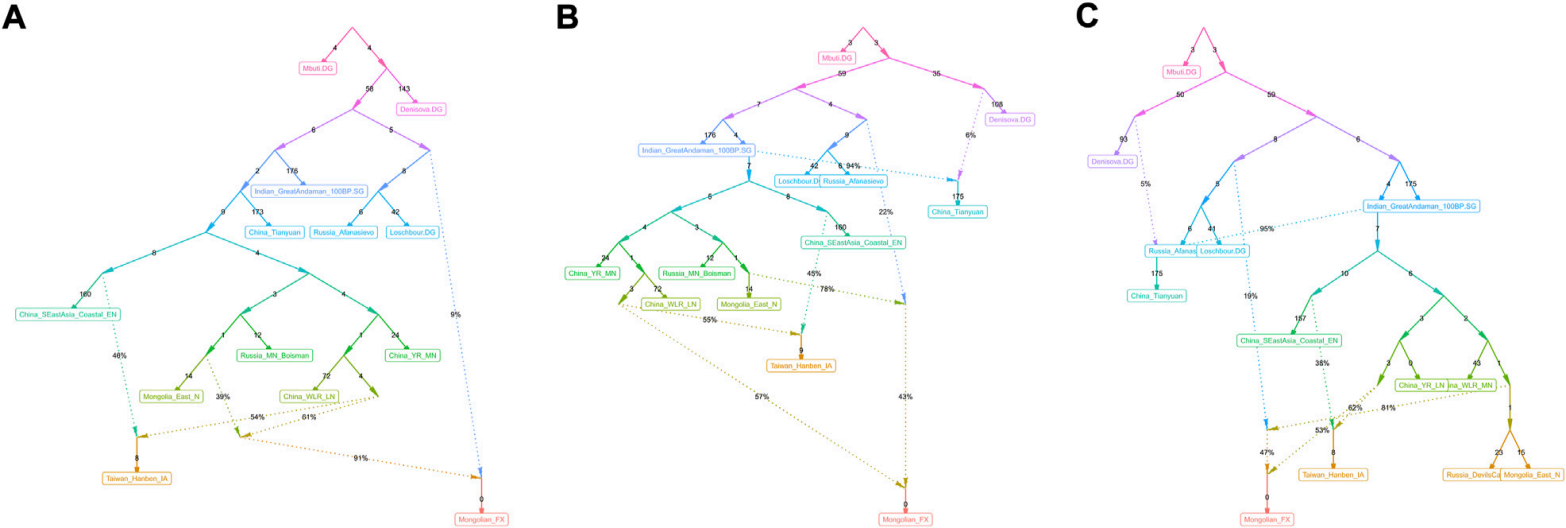
*qp*Graph-based phylogeny showed the genetic formation of modern Mongolians. **(A∼C)** Different genetic admixture models showed genetic formation of Fuxin Mongolians.

We used *qp*Graph and TreeMix methods with gene flow events to further explore possible ancestral sources and potential admixture signals and reconstruct deep phylogenetic structures in Fuxin Mongolians. In the *qp*Graph-based phylogenetic framework, we used Mbuti, Denisovan, Loschbour, GreatAndaman, and Tianyuan to construct the basal model. Neolithic hunter–gatherers in the Mongolian Plateau and Amur River Basin, millet farmers related to Yangshao and Lower Xiajiadian cultures in Yellow River and West River basins, Neolithic Qihe, Iron-Age Hanben, and Bronze-Age Afanasievo pastoralists were used as different ancestral source proxies (Figure 9). We found that Fuxin Mongolians could be modeled as the mixture of Neolithic millet farmer-related ancestry (53%–57%), Neolithic MP and ARB hunter–gatherer-related ancestry, and Western Eurasian-related ancestry (43%–47%), and we also found that Western Eurasian-related ancestry possessed 9% in the gene pool of Fuxin Mongolians. Our *qp*Graph models were consistent with the *qp*Adm results and further indicated that millet farmers and ARB and MP hunter–gatherers were the dominant ancestral sources and played an indispensable role in the genetic formation of modern Mongolians, and there was a limited genetic influence of Western Eurasian populations in Fuxin Mongolians.

**FIGURE 10:**
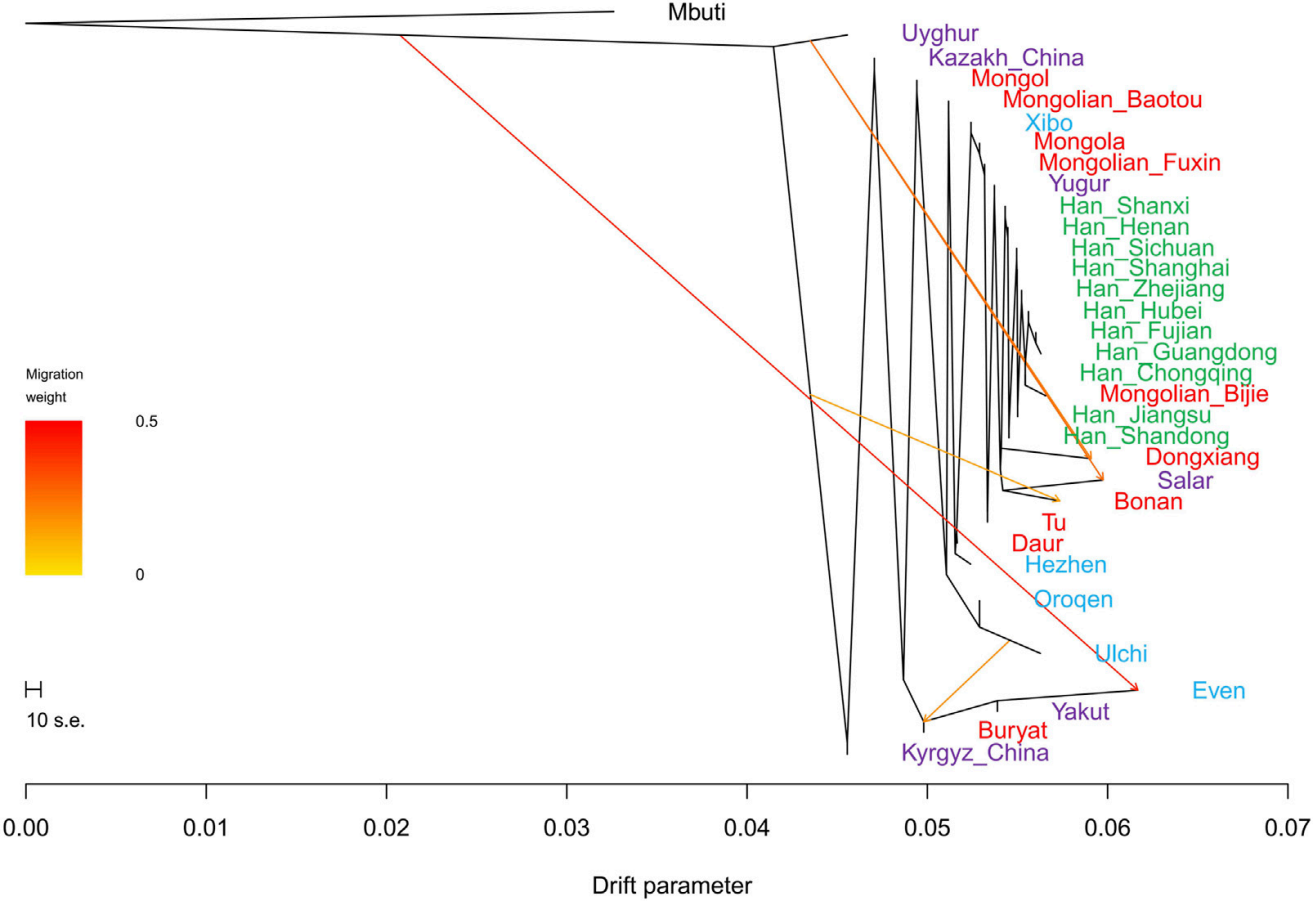
TreeMix-based maximum-likelihood phylogenetic tree with four migration events showed the population relationship among Han people and Turkic, Tungusic, and Mongolic speakers. Different colors represent different language categories.

In the TreeMix analysis (Figure 10), there was no significant gene flow event that could be found in Fuxin Mongolians. We found that Fuxin Mongolians clustered together with Mongolians from Hulunbuir and Baotou, Xibo, and Yugur. There was an obvious Han people cline, and Bijie Mongolians clustered with the Southern Han people. These results indicated that although there was an obvious genetic influence of the Han people on Fuxin Mongolians, and Fuxin Mongolians showed more significant genetic similarity with Northern Chinese Mongolians, which revealed genetic differences between Mongolians and Han people.

### ALDER and uniparental haplogroups

We next applied the ALDER method based on weighted linkage disequilibrium statistics to estimate the admixture time and explore possible admixture signals. There were multiple sources of admixture signals such as Han, Tungusic speakers, Mongolic speakers, Turkic speakers, and populations who harbored Western Eurasian-related ancestry. The results showed that the obvious eastern–western Eurasian population interaction and admixture occurred during a historic period (∼600–∼1,300 years ago) which was associated with the prosperity of the Silk Road and the westward expansion of the Mongol Empire (Supplementary Table S10). The time of admixture signals about the Han people could be dated back to ∼400–∼1,300 years ago which is approximately from the Tang dynasty to the Ming dynasty. The time of admixture signals about Western Eurasians could be dated back to about ∼600–∼1,500 years ago. Also, the Turkic- and Tungusic-related ancestries flowed into the gene pool of Fuxin Mongolians from ∼500 to ∼1,800 years ago.

In this study, we successfully identified paternal and maternal lineages in Fuxin Mongolians; 13 paternal haplogroups and 36 maternal haplogroups are listed in the Supplementary Material (Supplementary Table S11). We found that D5a2a, G1c, and R9c1a were the most frequent maternal haplogroups, and D1a1a1a1a2a and N1a2b1b1 were the dominant paternal haplogroups.

## Discussion

In this study, we performed a comprehensive population genetic analysis to explore the genetic structure and admixture history of the Mongolian population in Liaoning Province based on newly generated genome-wide SNP data. The results of the descriptive analysis (PCA, FST, and admixture) indicated that the genetic structure was consistent with the language category and geographical distribution, and populations from the same language group or adjoining geographic position had a close genetic affinity with East Asia. Fuxin Mongolians showed genetic similarity with the Mongolian population in Inner Mongolia (Hulunbuir and Baotou) and other Mongolic speakers such as Daur, Tu, and Bonan. Also, there were close genetic relationships between Fuxin Mongolians and Han people, Tungusic speakers (Hezhen and Xibo), and other geographically adjoining populations. Outgroup-*f*_*3*_ statistics further supported the aforementioned conclusions. We found that Fuxin Mongolians shared more genetic drifts with the Han people than other East Asians, and *f*_*4*_-statistics results further indicated the genetic similarity between Fuxin Mongolian and Han people, with insignificant Z-scores in the form of *f*_*4*_ (Han, Mongolian_Fuxin; Modern Eurasian population, Yoruba). Also, Fuxin Mongolians also shared alleles with Tungusic and Mongolic speakers which are supported by the results of Outgroup-*f*_*3*_ (Mongolian_Fuxin, Modern Population; Yoruba) and *f*_*4*_ (Hezhen/Xibo/Mongolia/Daur, Mongolian_Fuxin; Modern population, Yoruba). The results of admixture-*f*_*3*_ (Tungusic/Mongolic/Turkic speakers, CHS/CHB; Mongolian_Fuxin) which have a significant Z-score further indicated that Han people and Altaic speakers played an indispensable role in the formation of genetic makeup in Fuxin Mongolians. In addition, we also found the genetic contribution of the Western Eurasian population in Fuxin Mongolians. The admixture-*f*_*3*_ results showed that Fuxin Mongolians could be modeled as the mixture between Han people and Western Eurasian populations, and the ALDER-based admixture time revealed that the eastern–western Eurasian population admixture occurred during ∼600–∼1,300 years ago which is the period from the Tang dynasty to the Ming dynasty. The Silk Road and Maritime Silk Route were further developed during the Tang Dynasty; the Mongol Empire rose and expanded westward in the 13th century, and ancient Chinese navigators explored the ocean during the Ming dynasty. These events promoted prosperous economic, political, and cultural interactions and genetic exchanges between western and eastern Eurasia (Yao et al., 2004; Jeong et al., 2020). Also, due to special geographical location, Mongolians have become witnessers and promoters of the exchange and admixture of eastern–western Eurasia. The mixed pattern of eastern–western Eurasia is also revealed by mitochondrial and Y-chromosomal haplogroups. B4, D4, and R9 were the main maternal lineages, and O2a1, O2a2, and D1a were the dominant paternal lineages. These haplogroups have a high frequency in East Asian populations such as the Han people (Kivisild et al., 2002; Yao et al., 2002; Lang et al., 2019; Wang et al., 2021b), and Western Eurasian-specific haplogroups U4 also existed in Fuxin Mongolians (Melchior et al., 2010; Soares et al., 2010). Also, except East Asian-dominant haplogroups, there were Siberia-related haplogroups such as C7, N1a, N1b, and Q1a (Malyarchuk and Derenko, 2009; Stoneking and Delfin, 2010; Malyarchuk et al., 2011; Wang et al., 2021b), and these haplogroups further indicated the genetic contribution of Northern Asian populations in the genetic makeup of Fuxin Mongolians. We also found there were genetic differences among Mongolians, Fuxin Mongolians had less ARB and MP hunter–gatherer-related ancestry and Western Eurasian-related ancestry relative to Baotou Mongolian and Outer Mongolian, and Bijie Mongolians in Guizhou Province had more Sino-Tibetan-related ancestry and Southern-related ancestry than other Mongolian people. These results further supported the conclusions of previous studies (He et al., 2021; Yang et al., 2021).

Paleogenomic studies demonstrated that ancient Mongolians at different times and geographic regions exhibited disparate genetic profiles. There was a dynamic demographic history in the Mongolian Plateau, and multiple populations contributed to genetic ancestries to shape the genetic differences of ancient Mongolians: the main ancient Northeast Asian ancestry, the ephemeral ancient North Eurasian ancestry, the limited genetic contributions of Western Eurasian Steppe pastoralists and Iranian farmers, and recent genetic influence of the Han people (Jeong et al., 2020; Wang et al., 2021a). Our research indicated that these ancestries were still retained in present-day Fuxin Mongolians. The results of outgroup-*f*_*3*_ (Mongolian_Fuxin, Ancient population; Yoruba) and *f*_*4*_ (Ancient population1, Ancient population2; Mongolian_Fuxin, Yoruba) indicated that Fuxin Mongolians shared more genetic drifts and alleles with ancient YRB, WLRB, ARB, and MP populations. There were insignificant Z-scores in the form of *f*_*4*_ (YRB/WLB/ARB/MP, Mongolian_Fuxin; Ancient population2, Yoruba). The genetic contribution of ancient Mongolians also existed in the Fuxin Mongolian gene pool, Mongolia_8_EIA, Ulaanzukh_2_LBA, SlabGrave_1_EIA, Xianbei_IA, Xiongnu_7_EIA, CenterWest_4_LBA, Ulgii_1_EBA, and Khovsgol_6_LBA shared alleles with Fuxin Mongolians, and the results of *f*_*4*_-statistics in the form of *f*_*4*_ (Ancient Mongolian, Mongolian_Fuxin; Ancient population2, Yoruba) revealed that Fuxin Mongolians received more genetic influences of Mongolia_8_EIA and Ulaanzukh_2_LBA. According to historical records, modern Mongolians in Liaoning are mainly descended from two ancient Mongolian tribes, the “Mongolia Zhen” tribe, and the “Harqin” tribe. Fuxin Mongolians belong to the “Mongolia Zhen” tribe which has a history of more than 1,200 years, it originated in the Orkhon River Basin and Selenga River Basin, and this tribe expanded westward into the regions of Central Asia and Xinjiang with the development of strength. From the late 15th century to the early 16th century, the “Mongolia Zhen” tribe migrated to Hetao Plain in the Yellow River Basin and became an alliance with the “Tumet” tribe. In the 16th century, the “Tumet-Mongolia Zhen” tribe migrated eastward, and they finally settled in the western regions of Liaoning Province. The complex migration and admixture history shaped genetic differences between Fuxin Mongolians and other Mongolians. The admixture-*f*_*3*_ (Ancient population1, Ancient population2; Mongolian_Fuxin) results exhibited that Fuxin Mongolians could be modeled as the mixture between Neolithic and Iron–Age YRB and WLRB populations and Eurasian Steppe pastoralists, ancient Mongolians, and ancient Iranian. The models of the population mixture based on the *qp*Adm method showed YRB and WLRB millet farmers and ARB and MP hunter–gatherers were the dominant ancestral contributors, and there were additional gene flows related to Eurasian Steppe pastoralists and Iranian farmers. The *qp*Graph-based phylogenetic framework further supported the aforementioned results. The millet farmer-related ancestry was 53%–57%, and the MP and ARB hunter–gatherer-related ancestry and Western Eurasian-related ancestry were 43%–47%. We also found the Western Eurasian-related ancestry possessed 9% in the gene pool of Fuxin Mongolians. Also, the *qp*Adm results also indicated genetic differences between Fuxin Mongolians and other Altaic language speakers, and we could find there were more YRB-, WLRB-, and ARB-related ancestries in Fuxin Mongolians, which suggested that Fuxin Mongolians were more influenced by the expansion of millet farmers and retained more genetic contribution of ARB hunter–gatherers. In general, we found there were dynamic demographic history, complex population admixture, and multiple sources of genetic diversity in Fuxin Mongolians.

## Conclusion

In this study, we reported genome-wide SNP data of Fuxin Mongolians in Liaoning Province. We applied typical and advanced population genetic analysis methods [principal component analysis (PCA), Admixture, FST, *f*_*3*_-statistics, *f*_*4*_-statistics, *qp*Adm/*qp*Wave, *qp*Graph, ALDER, and TreeMix] to explore genetic structure and admixture history of Fuxin Mongolians. We found that Fuxin Mongolians had a close genetic relationship with the Han people, northern Mongolians, other Mongolic speakers, and Tungusic speakers in East Asia. Also, we found that Neolithic millet farmers in the Yellow River Basin and West Liao River Basin and Neolithic hunter–gatherers in the Mongolian Plateau and Amur River Basin were the dominant ancestral sources, and there were additional gene flows related to Eurasian Steppe pastoralists and Neolithic Iranian farmers in the gene pool of Fuxin Mongolians. These results shed light on dynamic demographic history, complex population admixture, and multiple sources of genetic diversity in Fuxin Mongolians.

## Data Availability

The datasets presented in this study can be found in online repositories. The names of the repository/repositories and accession number(s) can be found below: https://zenodo.org/record/6839600, doi: 10.5281/zenodo.6839600.
