# Supplementary figures and images for: Genomic insights into the genetic structure and population history of Mongolians in Liaoning Province
Source: Front Genet. 2022 Oct 12;13:947758. doi: 10.3389/fgene.2022.947758 (PMC9596793; doi:10.3389/fgene.2022.947758)

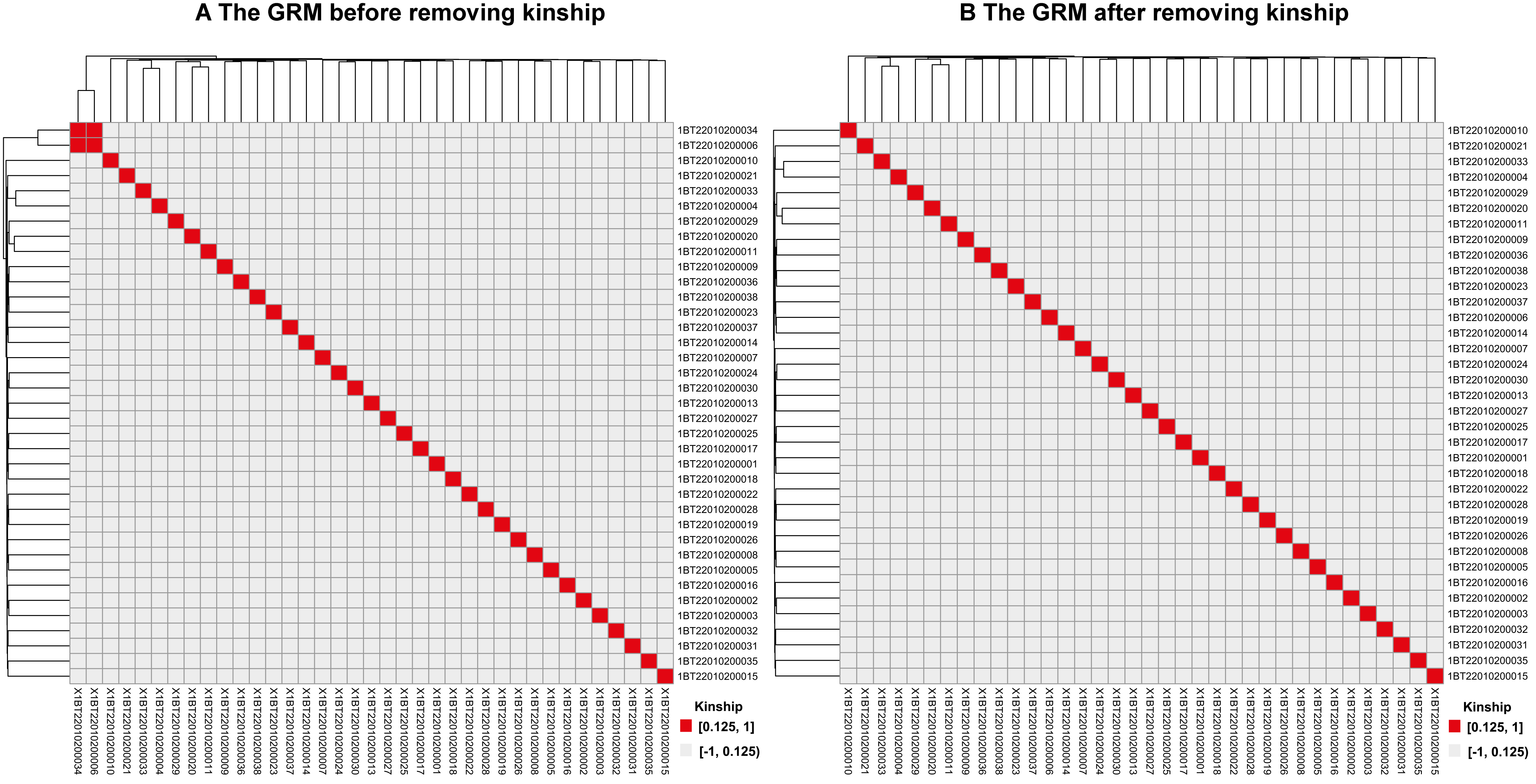

Supplement: Supplementary file 4 [file Image1.TIF]
